# Does Pre-Acclimation Enhance the Tolerance of *Quercus ilex* and *Arbutus unedo* Seedlings to Drought?

**DOI:** 10.3390/plants14030388

**Published:** 2025-01-27

**Authors:** Angela Balzano, Chiara Amitrano, Carmen Arena, Antonio Pannico, Rosanna Caputo, Maks Merela, Chiara Cirillo, Veronica De Micco

**Affiliations:** 1 Department of Wood Science and Technology, Biotechnical Faculty, University of Ljubljana, Rožna Dolina, Cesta VIII/34, 1000 Ljubljana, Slovenia; angela.balzano@bf.uni-lj.si (A.B.); maks.merela@bf.uni-lj.si (M.M.); 2 Department of Agricultural Sciences, University of Naples Federico II, Piazza Carlo di Borbone 1, 80055 Portici, Italy; chiara.amitrano@unina.it (C.A.); antonio.pannico@unina.it (A.P.); rosanna.caputo@unina.it (R.C.); 3 Department of Biology, University of Naples Federico II, Via Cinthia 21-26, 80126 Napoli, Italy; c.arena@unina.it

**Keywords:** compartmentalization, drought stress, pinning, plant functional traits, wood production, xylem plasticity

## Abstract

Mediterranean forests are severely threatened by increasing seedling mortality due to harsh environmental conditions, especially drought. In this study, we investigate whether seedlings of *Quercus ilex* and *Arbutus unedo*, previously exposed to water deficit, acquired tolerance to summer drought. Seedlings of the two species were grown from April to September in a plastic tunnel greenhouse and exposed to two irrigation regimes (control, 100% water holding capacity; water-stressed, 50% of control). In mid-August, the irrigation of all plants was suspended for three weeks. The response of the species was analyzed to evaluate survival, growth, ecological, and anatomical traits of wood produced under stressful conditions and marked through the pinning technique. The results suggest that both species show pre-acclimation to drought, with *Q. ilex* demonstrating a marked increase in survival percentage. This is likely due to a reduction in vessel size in response to previous water stress. In contrast, in *A. unedo*, the higher frequency of narrower vessels allowed safer water transport compared to *Q. ilex*, thus explaining the slight increase in survival. Overall results indicated that the two species adopt different strategies to overcome drought, providing valuable insights for managing seedlings in natural ecosystems and urban green spaces.

## 1. Introduction

The establishment and success of plant populations in Mediterranean forests are severely threatened by seedling mortality, which is exacerbated by the harsher conditions resulting from climate change [1]. The frequency of natural extreme events such as heat waves, flooding, and fire in the Mediterranean basin has increased in the last decades and is expected to worsen [2]. While many Mediterranean shrub and tree species can adjust their physiology and develop structural traits related to plant hydraulics to adapt and survive under limiting conditions in the adult phase, seedlings and young plants are more vulnerable to environmental stressors, and particularly exposed to drought-induced mortality caused by extreme events [1,3]. Therefore, the regeneration of populations of some species at the semi-arid edge of their territory is threatened, as recently reported for *Quercus ilex* L. [4]. Seedling death during drought results from a complex interplay of physiological and physical responses, including stomatal closure, cavitation, dehydration, tissue damage, and limitations in photosynthesis. Together, these factors contribute to the susceptibility and death of seedlings under drought conditions [5]. Moreover, vulnerability to cavitation plays a key role in determining seedling drought tolerance and survival in Mediterranean areas [1,3,6]. Some plant species possess intrinsic stress-protective systems based on stress memory mechanisms, meaning the that exposure to a stressor enhances the tolerance to subsequent stress events [7,8]. Different types of stress memories have been described for model species and recently investigated in conifers, but these mechanisms are still not completely understood [9]. Epigenetic mechanisms for stress memory and adaptation have also been studied in *Populus* and *Q. ilex*, where changes in the methylation pattern of DNA were observed under drought conditions [10].

For perennial forest species, the occurrence of stress-protective systems would be very important in the sight of climate change, since they are exposed to multiple stressors during their lifetime that are expected to increase (e.g., drought, high temperature, extreme events). Although the mechanisms are not yet fully deciphered, pre-acclimation is considered crucial to improve the seedlings’ resilience and enable them to better adapt to specific environmental stresses when exposed to natural conditions [11]. Indeed, in grass species, the exposure of seedlings to controlled stress conditions before they are exposed to the natural environment has been shown to pre-acclimate them to drought stress by stimulating physiological, morphological, and biochemical adjustments [12]. Pre-acclimation is also widely used to reduce agricultural water use by improving plant access to existing soil water and by mitigating yield losses to drought in seasonally water-limited environments [13].

Growth experiments in controlled or semi-controlled environments allow us to better understand the effects of pre-acclimation, which can involve the adjustment of plant structural hydraulic traits. Analyses of the wood structure provide important information about trees’ growth and physiological response to their environment. Woody species growing in Mediterranean sites are expected to have unique patterns of xylogenesis resulting in a start-and-stop wood formation leading to intra-annual density fluctuations (IADFs) in tree rings, thus forming sectors of wood optimized to transport water according to intra-seasonal fluctuating water availability [14]. The formation of IADFs in tree rings is considered a sign of plasticity and, in seedlings subjected to a mild water deficit, may be a strategy to pre-acclimate the plant to subsequent stress events. Indeed, IADF occurrence would increase the variability of xylem hydraulic traits within a growth ring, thus ensuring sectors of the ring either favoring the safety or the efficiency of water transport, depending on the intra-seasonal water availability. The correct interpretation of IADFs is necessary for climatic reconstruction and for predicting wood growth responses to expected climate changes, since their occurrence is linked to the complex interactions among plant physiological processes (e.g., photosynthetic efficiency, resources allocation, relations with primary growth) and both intrinsic factors and environmental factors during wood formation [15].

Considering the above, this study aims to evaluate the effect of a drought event on *Quercus ilex* L. (holm oak) and *Arbutus unedo* L. (strawberry tree) seedlings previously subjected to two regimes of water availability (control—C and water-stressed—WS). We hypothesize that seedlings subjected to pre-acclimation (WS) may better adapt to the subsequent water deprivation. In particular, we analyzed plant survival, growth, ecological, and anatomical traits of the wood produced under the stressful water deprivation conditions to evaluate pre-acclimation strategies of the two species. More specifically, we assessed plants’ photosynthetic performance by measuring gas exchange and chlorophyll “a” fluorescence emission, also including leaf water potential to determine their water status. Since the two species are prone to form IADFs, the pinning technique was used to mark the reference point (by wounding the xylem tissue with a needle) on the stem before the water deprivation to observe the response of the xylem in terms of quantitative functional traits in the newly formed wood [16]. Understanding the occurrence of structural traits allowing pre-acclimation in the seedlings of Mediterranean species is of interest to evaluate their regeneration potential in a scenario of increasing frequency of water stress events.

## 2. Results

### 2.1. Plant Growth

**Figure 1:**
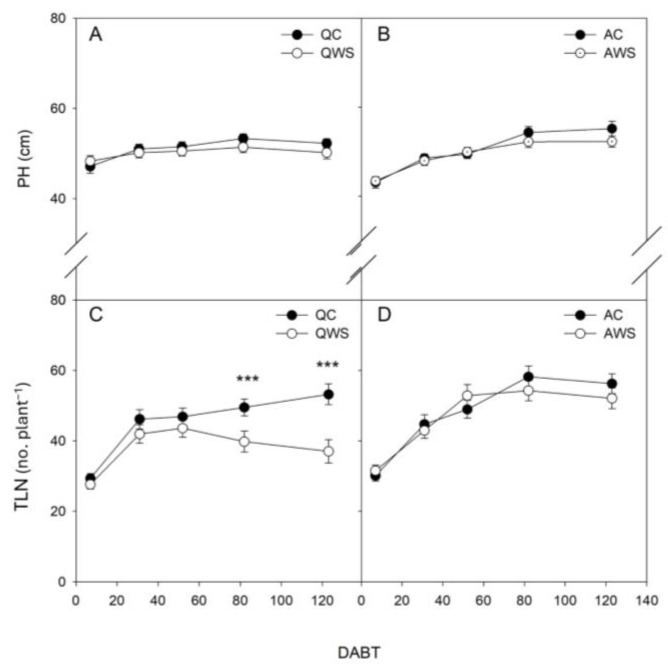
Plant height (PH) (**A**,**B**) and total leaf number (TLN) (**C**,**D**) in *Q. ilex* control (QC) and water-stressed (QWS) seedlings (**A**,**C**) and *A. unedo* control (AC) and water-stressed (AWS) seedlings (**B**,**D**). Mean values and standard errors are shown. DABT, Days After Beginning of irrigation Treatment. ***, indicate significant differences at *p* < 0.001.

**Figure 2:**
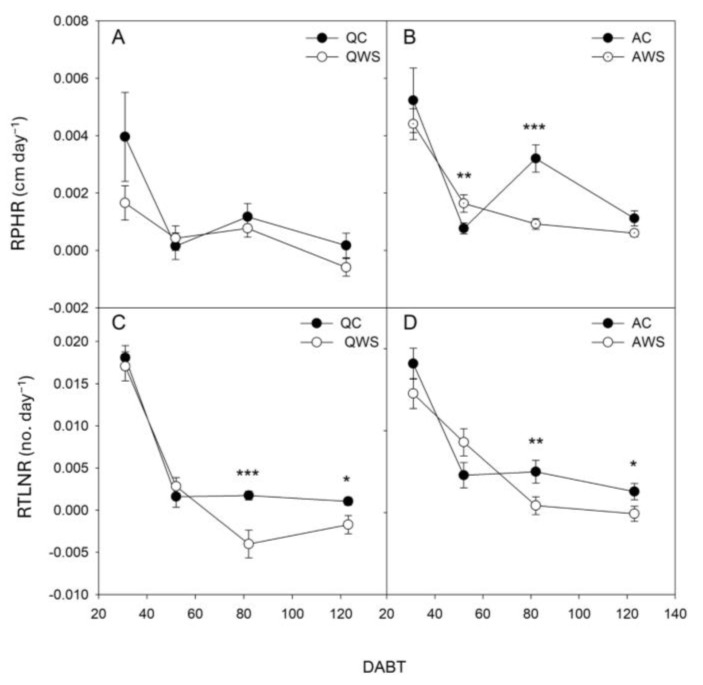
Relative plant height rate (RPHR) (**A**,**B**) and relative total leaf number rate (RTLNR) (**C**,**D**) in *Q. ilex* control (QC) and water-stressed (QWS) seedlings (**A**,**C**) and *A. unedo* control (AC) and water-stressed (AWS) seedlings (**B**,**D**). Mean values and standard errors are shown. DABT, Days After Beginning of irrigation Treatment. *, **, and ***, indicate significant differences at *p* < 0.05, 0.01, and 0.001, respectively.

The seedlings that underwent a water stress pre-treatment (WS) showed higher survival after the water deprivation than control (C) seedlings. Specifically, the survival of *Q. ilex* control seedlings was 56%, which increased to 78% in WS seedlings. In *A. unedo*, the survival of control seedlings was nearly 80%, increasing to 87% in WS seedlings. Plant growth parameters of the surviving seedlings are reported in Table 1. The effects of both the main factors (species, S; treatment, T) were significant for total leaf number (TLN), while only the effect of T was significant for plant height (PH). Indeed, TLN was higher in *A. unedo* than in *Q. ilex* seedlings. In general, the WS treatment negatively affected the growth of the seedlings, reducing plant height (PH) and TLN by 4.3% and 18%, respectively (Table 1). Growth dynamics are shown in Figure 1 and Figure 2.

Growth dynamics differed between control (C) and water-stressed (WS) plants in both species (Figure 1 and Figure 2), with larger variations in the case of TLN (Figure 1C,D and 2C,D). Specifically, regarding plant height (PH), the relative plant height growth rate (RPHR; Figure 2A) showed similar patterns in *Q. ilex* for both C and WS seedlings, but WS exhibited less variation in growth with increasing DABT (Days After Beginning of irrigation Treatment). Differently, in *A. unedo*, there were greater differences between C and WS. Indeed, *A. unedo* C showed a recovery at 82 DABT, only to decline again to values similar to AWS at 123 DABT (Figure 2A,B).

In the case of relative total leaf number rate (RTLNR; Figure 2C,D) similar patterns between *Q. ilex* and *A. unedo* are shown. In particular, from 52 to 123 DABT, both QC and AC maintained similar values, always higher than QWS and AWS, which instead decreased as the DABT progressed.

### 2.2. Chlorophyll “a” Fluorescence Emission, Gas Exchange, and Leaf Water Potential

The outputs of chlorophyll “a” emission measurement, gas exchange, and leaf water potential are reported in Table 2. The effect of S and T as main factors was never significant for photochemistry parameters. However, their interaction (S × T) was significant for ΦPSII, with values in *A. unedo* C significantly higher than in *Q. ilex* C, while intermediate values were recorded in *Q. ilex* WS and *A. unedo* WS. The interaction was also significant for NPQ, with *Q. ilex* C and *A. unedo* WS achieving values significantly higher than *A. unedo* C. Concerning gas exchange parameters, only T as the main factor showed a significant effect for P_N_, g_s_, and T_R_, with significantly higher values in control than water-stressed seedlings (about −50%). In T_R,_ S also elicited a significant effect with higher values in *A. unedo* compared to *Q. ilex*. The T × S interaction was not significant for gas exchange parameters. The Ψ_l_ was higher in *Q. ilex* compared to *A. unedo*, with no significant differences between C and WS. However, the interaction resulted in significantly higher values in *Q. ilex* WS than *A. unedo* WS and *A. unedo* C, with *Q. ilex* C showing intermediate values.

### 2.3. Wood Production, Growth Increments, and Wood Anatomical Traits

**Figure 3:**
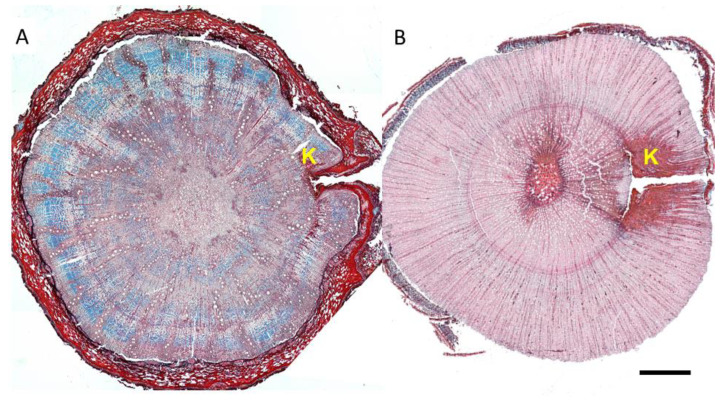
Light microscopy views of cross-sections of *Q. ilex* (**A**) and *A. unedo* stems (**B**) showing wounded wood and callus lip formation (K) at the pinning point level. Images are at the same magnification. Scale bar = 250 µm.

Upon analysis of cross-sections, both species exhibited the typical structure of juvenile wood of diffuse-porous species, with a high frequency of vessels and rays. The pinning wound, which served as a marker for the wood formed after the start of the experiment, was visible in the cross-sections (Figure 3).

**Figure 4:**
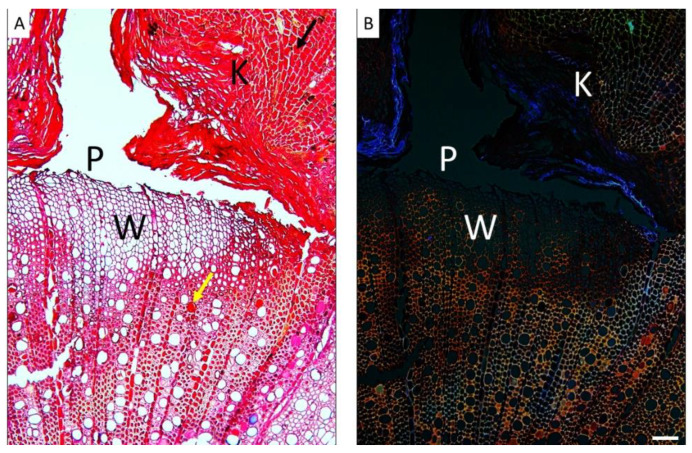
Light (**A**) and epi-fluorescence (**B**) microscopy views of cross-sections of *A. unedo* stems showing the wounded wood (W) and callus lip formation (K) at the pinning point level (P). (**A**) Deposition of phenolic compounds and other organic occlusions in the parenchyma cells (black arrow) and conduits (yellow arrow). Suberization of cell walls is visible in blue (**B**). Images are at the same magnification. Scale bar = 100 µm.

The sections corresponding to the pinning zone in both species showed the typical anatomical reaction to the mechanical wounding caused by the needle puncturing the cambium (Figure 3 and Figure 4). More specifically, the pinning hole in the bark was visible, as well as the zone of callus lip formation, where cells hyper-proliferate to close the scar, show lignification and suberization of cell walls, and accumulate occlusions to develop the so-called barrier zone (Figure 3 and Figure 4).

In both species, cambium activity was restored, thus forming xylem cells to close the wound. The compartmentalization zone was visibly demarcated in the cross-sections of both species. However, seedlings of *A. unedo* exhibited a more evident reaction response than *Q. ilex* to the pinning wound, with a more extended area characterized by the deposition of phenolic compounds and other organic occlusions in parenchyma cells, fibers, and conduits.

**Figure 5:**
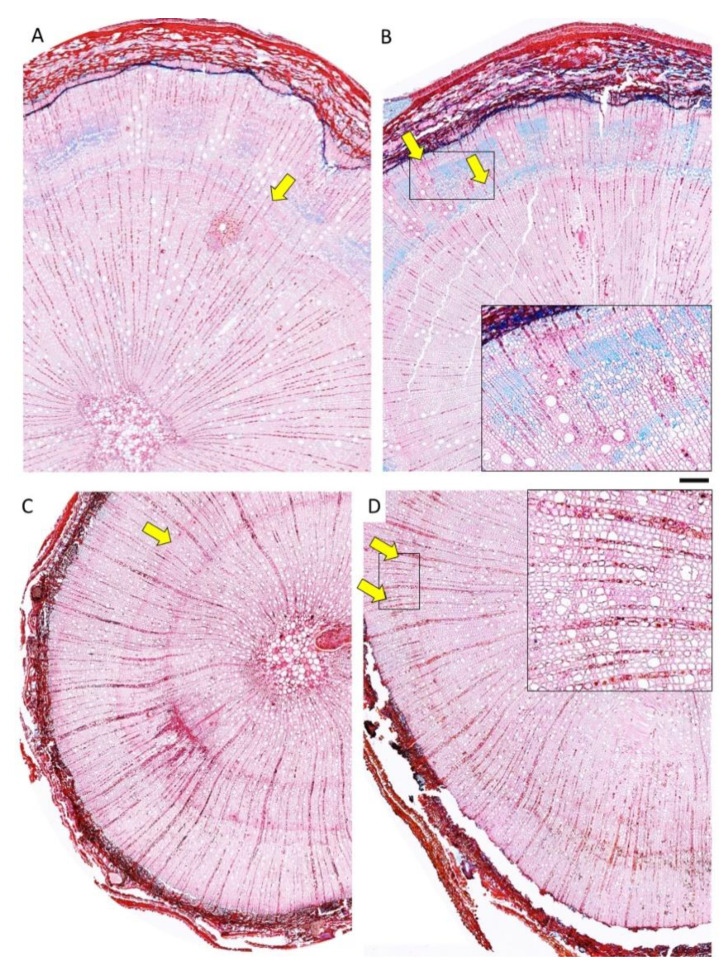
Light microscopy views of cross-section showing IADFs (yellow arrows) in stems of *Q. ilex* control (**A**) and water-stressed (**B**) and *A. unedo* control (**C**) and water-stressed (**D**) seedlings. Images are at the same magnification. Scale bar = 250 µm. Higher magnification details are in selected areas with black borders.

Concerning the anatomical traits (Table 3), the effect of the species (S) was significant in all analyzed parameters. *A. unedo* seedlings exhibited significantly wider new wood increments than *Q. ilex* ones, as well as a higher number of IADFs (Figure 5). They also showed a significantly higher frequency of narrower vessels (i.e., VLA) than *Q. ilex*, and an overall higher WTA. The effect of T was significant only in the case of VLA, which showed significantly higher values in WS seedlings. The interaction S × T was significant in terms of VLA, VF, and WTA. *Q. ilex* C showed significantly higher values for VLA than *Q. ilex* WS, which in turn had higher values than *A. unedo* C and *A. unedo* WS. VF and WTA were significantly higher in *A. unedo* C than in *Q. ilex* in both water regimes (*Q. ilex* C and *Q. ilex* WS).

## 3. Discussion

Moderate environmental stresses are recognized to enhance tolerance to a secondary stress in a phenomenon referred to as training, hardening, imprint, priming, conditioning, or acclimation [10]. This might be a fundamental phenomenon, possibly counteracting seedling mortality in ecosystems threatened by climate change. In this study, seedlings of *Q. ilex* and *A. unedo* subjected to a mild water deficit (pre-acclimation) showed enhanced survival after exposure to severe stress caused by successive water deprivation, probably in combination with high temperatures during the summer season. The survival of pre-acclimated water-stressed seedlings, compared to not-stressed controls, increased more in *Q. ilex* than in *A. unedo* seedlings, whose survival was already much higher than *Q. ilex* under control conditions. The higher potentiality of survival in oak during drought compared to *A. unedo* may be likely due to differences in hydraulic traits and wood anatomy, since no difference in photosynthetic rate (P_N_), stomatal conductance (g_S_), and photochemical efficiency was found between the two species. During the pre-acclimation period, we observed a decrease in gas exchanges, specifically, a reduction in P_N_, g_S_, and transpiration rate (T_R_) in water-stressed plants compared to control ones. This is also in line with the plant growth analyses. Indeed, starting from about 50 DABT, both plant height and number of leaves growth rates were mostly lower under water stress.

The photosynthesis down-regulation may be a response to hydraulic limitations likely imposed by xylem structures, which regulated their vulnerability to embolism [17,18,19]. Despite the reduced gas exchanges under water scarcity, the functionality of the photosystem remained intact for both species. This was confirmed by the Fv/Fm ratio, which showed no significant changes between stressed and control plants. We hypothesize that such a regulation observed in the pre-acclimation period may also be maintained during the three-week drought event, favoring the safety of the photosynthetic apparatus.

In terms of wood anatomical traits, *A. unedo* is considered a very plastic species, as it is very prone to forming IADFs. Under fluctuating environmental conditions, this species can form several growth rings per calendar year, having a start-and-stop cambial activity that allows the plants to benefit from even short periods of favorable growth conditions [14,20,21,22]. Compared to *Q. ilex*, *A. unedo* showed a higher number of IADFs and an overall more plastic response at the xylem level after mechanical wounding (i.e., growth reaction and compartmentalization after pinning), while water stress did not significantly alter parameters such as vessel lumen size. On the other hand, *Q. ilex* showed a lower tendency to form IADFs and less responsiveness to wounding, while the reaction to water stress was quite severe in terms of vessel size, with VLA reduced by about 25% in WS seedlings compared to controls. Whether the formation of IADFs in Mediterranean species is a sign of acclimation or vulnerability to drought conditions is still under debate [15,23]. However, species forming IADFs can benefit from short periods of favorable conditions under fluctuating environments, maintaining active growth or restoring it. A recent study collecting data on IADF frequency in adult plants of several softwood and hardwood species across Europe suggested that trees showing bimodal growth patterns will likely form more IADFs as an adaptation to increasing temperature and drought [23]. In our study, IADF frequency was not increased in water-stressed seedlings, suggesting that the imposed water stress conditions did not alter cambium production regarding cell division. The subsequent phases of xylogenesis were influenced in *Q. ilex*, where the dynamics of cell enlargement and cell wall deposition resulted in narrower vessels in stressed seedlings. The lower aptitude to form IADFs in *Q. ilex* compared to *A. unedo* is in line with previous findings in adult plants. In *Q. ilex* trees, increasing temperature has been reported to limit the formation of IADFs, likely due to an early stop of cambial activity, which is not easily resumed if environmental conditions ameliorate [24]. Therefore, *Q. ilex* would react to water stress by adopting a strategy based on the reduction of vessel size, favoring water transport safety against embolism. Since Carlquist’s early studies on ecological wood anatomy, the relation between vessel size and vulnerability to embolism has been debated [25]. Vessel size alone is not responsible for xylem vulnerability, the latter also being dependent on other traits such as frequency distributions of all diameter classes, vessel network connectivity, and pitting [26,27,28]. However, vessel size is strongly correlated with the pressure at which 50% loss in hydraulic conductivity occurs (P50), with narrower vessels being less efficient but safer than larger ones [28]. The narrower vessels in *A. unedo*, accompanied by higher frequency, allowed a larger water transport area characterized by high safety compared to *Q. ilex*. This would explain why the survival in the first species was already high in control seedlings, suggesting no need to adjust xylem traits to further improve safety. On the other hand, *Q. ilex* seedlings adopted a strategy of increasing the number of narrower vessels under water stress conditions, which allows for maintaining the same but safer water transport area as control seedlings. The safer wood in WS *Q. ilex* seedlings would allow slow but continuous water flow under water stress, which also allows keeping stomata open at more negative water potential.

Both species respond to water stress by closing stomata, thus reducing the water loss by transpiration. Stomatal limitations, carried out to save water, are responsible for the decline in net photosynthesis. The stomatal closure to reduce transpiration under water stress conditions is the typical behavior of plants with C3 photosynthetic pathways: starving or thirsting under stressful conditions [29]. However, the limitation of carbon fixation in both species did not affect the photosystem functioning, as indicated by the quantum yield of PSII (ΦPSII) and electron transport rate (ETR), which did not change in control and stressed plants. In particular, the values of the maximum PSII photochemical efficiency (Fv/Fm), close to those of fully healthy plants, indicated the lack of damage to photosystems [30]. Contrary to *Q. ilex*, the interaction S × T revealed differences between *A. unedo* unstressed and stressed plants in non-photochemical processes. The non-photochemical quenching (NPQ) is a crucial mechanism in plants’ PSII photoprotection against environmental stress. It is important to note that elevated NPQ values are typically linked to conformational changes within the PSII photosystem, enabling the dissipation of excess excitation energy as heat in a harmless manner [31]. Our study revealed that water stress in the pre-acclimation period induces an increase in NPQ in *A. unedo*, suggesting that this species has a robust intrinsic capability to dissipate excess light energy as heat when carbon fixation is limited. In contrast, the photosynthetic apparatus of *Q. ilex* did not exhibit this adaptation, making it more susceptible to photodamage under prolonged stress conditions [32].

The overall analysis suggested that in both species, the water stress pre-acclimation treatment mitigated the effects of the subsequent drought event, but with different degrees of benefit due to their different wood anatomy. Although other factors (such as the resource allocation between below- and above-ground organs, leaf venation, etc.) influence the degree of acclimation, data suggest that the safer wood traits and higher cambium plasticity (i.e., high aptitude to form IADFs) of *A. unedo* seedlings explain why the survival of control plants was not much increased in water-stressed plants, as if its traits were already designed to withstand severe drought conditions. Additionally, in terms of reaction to mechanical wounding, the more evident response of *A. unedo* compared to *Q. ilex* may give it a competitive advantage in challenging environments. The formation of a compartmentalization zone is a common mechanism in trees to limit the spread of pathogens, insect attacks, and decay. Therefore, this defense mechanism is critical for the survival and longevity of trees [33,34]. On the other hand, in *Q. ilex*, the pre-acclimation of seedlings with mild water stress was helpful since the narrower vessels developed in water-stressed plants prevented the embolism phenomena, thus improving survival after the drought event.

The different efficacy of pre-acclimation in the various Mediterranean species should be studied because they can influence forest regeneration and provide useful information in forest management strategies.

## 4. Materials and Methods

### 4.1. Plant Material, Experimental Conditions, and Treatments

**Figure 6:**
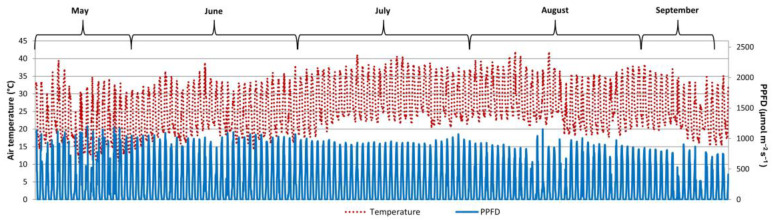
Temperature and photosynthetic photon flux density (PPFD) recorded during the experiment.

This experiment was carried out under a plastic tunnel solar greenhouse at the Department of Agricultural Sciences, University of Naples Federico II in southern Italy. One-year-old seedlings from rooted cuttings of *Quercus ilex* (Q, holm oak) and *Arbutus unedo* (A, strawberry tree) were obtained from the nursery Vivaio Garden Forest (Maddaloni, Caserta, Italy). The plants (66 per species, homogeneous in terms of morphological development) were root ball transplanted in 15 cm diameter (2.5 L) pots in late April and, after ten days, subjected to two different irrigation treatments. The first treatment provided 100% water holding capacity (control, C), while the second treatment provided 50% reintegration of water (water-stressed, WS). The plants were irrigated daily; the daily water use of the plants was calculated to determine the amount of water to be applied per treatment. More specifically, per each species, 6 seedlings were placed on an electronic balance at the same plant density (10 seedlings/m^2^); the pots were covered with plastic film to avoid evaporation from the soil, and transpiration was determined by a gravimetric method [35]. Air temperature and photosynthetic photon flux density (PPFD) during the entire experimental trial were monitored and recorded (WatchDog, Spectrum Technologies Inc., Aurora, IL, USA) (Figure 6). The plants were arranged in a randomized complete block design with 6 groups of 5 plants per treatment per each species (6 groups × 5 plants × 2 treatments × 2 species). In August, 90 days after the beginning of irrigation treatments (DABT), the irrigation was suspended for 21 days to simulate a drought event and later resumed until the end of the experiment in September (at 123 DABT).

### 4.2. Plant Growth

During the cultivation cycle, plant growth was monitored by recording plant height (PH) and total leaf number (TLN) on 10 plants per treatment per species, at 7, 31 52, 82, and 123 DABT.

The rate of increase in PH per unit of PH (relative plant height rate, RPHR) was calculated as follows:

RPHR = [ln(PH_2_) − ln (PH_1_)]/(t_2_ − t_1_)

where *t* is the treatment duration in days and subscripts are the timing of data collection.

The rate of increase in TLN per unit of TLN (relative total leaf number rate, RTLNR) was calculated as follows:

RTLNR = [ln(TLN_2_) − ln (TLN_1_)]/(t_2_ − t_1_)

where *t* is the treatment duration in days and subscripts are the timing of data collection.

### 4.3. Leaf Photosynthesis and Water Potential Monitoring

Leaf photosynthesis was assessed by means of gas exchange and chlorophyll *a* fluorescence emission measurements on youngest fully developed leaves at 84 DABT (just before the period of irrigation suspension) between 11:00 am and 13:00 pm, using six different leaves (six replicates) per treatment, as described in Ref. [35]. Stomatal conductance (g_s_), transpiration rate (T_R_), and net CO_2_ assimilation rate (P_N_) were measured using a portable gas exchange analyzer (LCA 4; ADC BioScientific Ltd., Hoddesdon, UK) equipped with a broad-leaf PLC (cuvette window area, 6.25 cm^2^). Ambient values for photosynthetically active radiation (PAR), relative humidity (RH%), and carbon dioxide concentration (CO_2_) were used, and the air flow rate was 400 mL s^−1^.

On the same leaves, fluorescence emission measurements were carried out in the light and in the dark with a portable FluorPen FP100max fluorometer equipped with a light sensor (Photon System Instruments, Brno, Czech Republic). A blue LED internal light of approximately 1–2 mol m^−2^ s^−1^ was used to generate the basal fluorescence signal (Fo) on 30’ dark-adapted leaves. A 1 s-long saturating light pulse of 3000 mol m^−2^ s^−1^ was used to generate the maximum fluorescence level in the dark (Fm). According to Refs. [36,37], the maximum photochemical efficiency of PSII (Fv/Fm) was determined as (Fm Fo)/Fm. The quantum yield of PSII electron transport (ΦPSII = (Fm’ − Fs)/Fm’) and the non-photochemical quenching (NPQ = (Fm − Fm’)/Fm) were calculated according to Ref. [38]. The electron transport rate (ETR) was calculated following the equation of Ref. [39].

On the same day (84 DABT), leaf water potential (Ψ_l_) was measured at midday (12:00 h) using the pressure chamber technique [40] on three well-lit leaves per plant from three plants per treatment. The precautions recommended by Ref. [41] were followed during measurements.

### 4.4. Wood Anatomical Analyses

At the start of the experiment (7 DABT), the cambium position was marked using the pinning method, which involves inserting a needle from the bark through the cambial zone into the xylem [42]. This method allowed us to visualize the cambium reaction and to identify the region of wood formed during the experiment by cutting sections at the injured wood region (pinned zone) at the end of the trial. Therefore, at the end of the experiment, stem sub-samples (1 cm long) were taken from four control and four water-stressed plants per each species, in the region where the pinning was performed. Sub-samples were stored in F.A.A. fixative solution (50% ethanol solution, 38% formaldehyde, glacial acetic acid, 90/5/5 by volume) for one week and then transferred to 70% ethanol, followed by dehydration in increasing ethanol concentration up to 100%, and infiltration with Bio-Clear (D-limonene) (Bio Optica, Milan, Italy) and paraffin for four hours in each one. Subsamples were embedded in paraffin blocks through a Leica TP1020-1 (Nussloch, Germany) tissue processor. Cross sections (9 µm) were cut by a semi-automatic rotary microtome RM 2245 (Leica), cleaned of residual paraffin by washing with Bio-Clear and ethanol, stained with an aqueous solution of safranin (0.04%) and astra blue (0.15%) [42], and permanently mounted in Euparal (Chroma3C-239Waldeck, Münster, Germany) on glass slides.

The slides were viewed by a Zeiss Axio Imager A.2 light microscope (Carl Zeiss Microscopy, White Plains, NY, USA), and images were captured with a Zeiss Axiocam 712 colour (Carl Zeiss Microscopy GmbH, Jena, Germany). Quantitative wood anatomy was performed using ImageJ software version 1.42 (http://rsb.info.nih.gov/ij/ accessed on 20 July 2024). The width of the wood formed from the beginning to the end of the experiment was identified, using the scar formed by the pinning as a mark. The width of the newly formed wood increments after pinning was measured (in 3 points per section). Within wood formed during the experiment (from the pinning point to the cambial zone), wood traits were quantified into three regions, including at least four rays in *A. unedo* and two radial vessel bundles in *Q. ilex.* Quantified parameters were vessel lumen area (VLA, µm^2^), vessel frequency (VF, n mm^−2^), determined by counting vessels in a known area [43], and water transport area (WTA, µm^2^ mm^−2^), determined as the sum of vessel lumen area in an area of 1 mm^2^. The wood rings that were developed after the pinning scar, if any, were defined as intra-seasonal growth flashes, thus Intra-annual Density Fluctuations (IADFs), given their formation within the timeframe of weeks, thus distinguishing them from typical annual (calendar) rings. The IADFs were counted for each stem.

### 4.5. Statistical Analysis of Data

Growth, leaf photosynthesis, water potential, and anatomical data were analyzed by two-way ANOVA, considering the species (S) and irrigation treatment (T) as main factors, as well as their interaction (S × T). To separate treatment means within each measured parameter, Duncan’s multiple range test was performed at a significance level of *p* ≤ 0.05. Shapiro–Wilk and Kolmogorov–Smirnov tests were performed to check for normality. Percent data were transformed through the arcsine function before statistical analysis.

## Figures and Tables

**Table 1:** Analysis of variance and comparisons of means for plant growth in terms of plant height (PH) and total leaf number (TLN) in *Q. ilex* (Q) and *A. unedo* (A) under the two treatments, control (C) and water stress (WS), at the end of the experiment (123 DABT). Results are reported as mean values ± standard error. Different letters correspond to significant differences according to Duncan’s multiple range test. NS, **, and ***, not significant or significant at *p* < 0.01, and 0.001, respectively.

	PH (cm)	TLN (n.)
Species (S)		
Q	51.21 ± 0.86 a	45.13 ± 2.01 b
A	53.61 ± 0.98 a	54.09 ± 2.31 a
Treatment (T)		
C	53.57 ± 0.91 a	54.65 ± 2.14 a
WS	51.25 ± 0.93 b	44.56 ± 2.20 b
Significance		
S	NS	**
T	**	***
S × T	NS	NS

**Table 2:** Analysis of variance and comparison of means for maximum PSII photochemical efficiency, (Fv/Fm), electron transport rate (ETR), quantum yield of PSII electron transport, (ΦPSII), non-photochemical quenching (NPQ), net CO_2_ assimilation (P_N_), stomatal conductance to water (g_S_), transpiration (T_R_), and leaf water potential (Ψ_l_) in *Q. ilex* (Q) and *A. unedo* (A) under the two control (C) and water stress (WS) treatments at 84 DABT. Results are reported as mean values ± standard error. Different letters correspond to significant differences according to Duncan’s multiple range test. NS, *, **, and ***, not significant or significant at *p* < 0.05, 0.01, and 0.001, respectively.

	F_v_/F_m_	ETR	ΦPSII	NPQ	P_N_ (µmol m^−2^ s^−1^)	g_s_ (mol m^−2^ s^−1^)	T_R_ (mol m^−2^ s^−1^)	Ψ_l_ (MPa)
Species (S)								
Q	0.77 ± 0.009 a	268.0 ± 27.9 a	0.43 ± 0.04 a	3.07 ± 0.53 a	3.73 ± 0.34 a	0.05 ± 0.007 a	1.21 ± 0.13 b	−2.94 ± 0.32 a
A	0.78 ± 0.001 a	314.2 ± 29.2 a	0.51 ± 0.05 a	2.26 ± 0.55 a	4.01 ± 0.33 a	0.06 ± 0.007 a	1.56 ± 0.12 a	−1.72 ± 0.32 b
Treatment (T)								
C	0.78 ± 0.002 a	296.4 ± 27.9 a	0.47 ± 0.04 a	2.60 ± 0.53 a	5.05 ± 0.32 a	0.07 ± 0.007 a	1.71 ± 0.11 a	−1.94 ± 0.31 a
WS	0.77 ± 0.002 a	283.2 ± 29.2 a	0.46 ± 0.06 a	2.77 ± 0.55 a	2.51 ± 0.35 b	0.03 ± 0.007 b	1.02 ± 0.13 b	−2.72 ±0.33 a
Interaction (S × T)								
QC	0.78 ± 0.008 a	232.5 ± 25.6 a	0.37 ± 0.04 b	4.07 ± 0.55 a	4.81 ± 0.59 a	0.06 ± 0.010 ab	1.43 ± 0.17 b	−2.43 ± 0.22 ab
QWS	0.77 ± 0.010 a	303.4 ± 50.4 a	0.48 ± 0.07 ab	2.09 ± 0.62 ab	2.29 ± 0.26 b	0.03 ± 0.009 b	0.91 ± 0.17 b	−3.45 ± 0.45 a
AC	0.79 ± 0.009 a	360.3 ± 26.5 a	0.58 ± 0.04 a	1.14 ± 0.44 b	5.33 ± 0.33 a	0.08 ± 0.007 a	2.01 ± 0.12 a	−1.45 ± 0.11 b
AWS	0.78 ± 0.010 a	259.0 ± 54.8 a	0.42 ± 0.09 ab	3.60 ± 1.37 a	2.69 ± 0.52 b	0.04 ± 0.011 b	1.11 ± 0.22 b	−1.99 ± 0.75 b
Sig.								
S	NS	NS	NS	NS	NS	NS	*	*
T	NS	NS	NS	NS	***	**	***	NS
S × T	NS	NS	*	**	NS	NS	NS	*

**Table 3:** Analysis of variance and comparison of means for wood increment width (WIW), IADF number, vessel lumen area (VLA), vessel frequency (VF), and water transport area (WTA) in *Q. ilex* (Q) and *A. unedo* (A) seedlings under the two treatments control (C) and water stress (WS). Results are reported as mean values ± standard error. Different letters correspond to significant differences according to Duncan’s multiple range test. NS, *, **, and ***, not significant or significant at *p* < 0.05, 0.01, and 0.001, respectively.

	WIW (mm)	IADFs (n.)	VLA (µm^2^)	VF (n/mm^2^)	WTA (µm^2^/mm^2^)
Species (S)					
Q	1.06 ± 0.13 b	0.58 ± 0.20 b	651.5 ± 9.65 a	36.90 ± 16.06 b	23,336 ± 5889 b
A	1.39 ± 0.09 a	1.19 ± 0.14 a	362.2 ± 9.23 b	121.6 ± 17.7 a	44,185 ± 6518 a
Treatment (T)					
C	1.23 ± 0.11 a	1.07 ± 0.17 a	556.3 ± 7.68 a	92.14 ± 14.58 a	40,251 ± 5349 a
WS	1.22 ± 0.11 a	0.70 ± 0.18 a	457.4 ± 10.92 b	66.37 ± 19.00 a	27,270 ± 6968 a
Interaction (S × T)					
QC	1.06 ± 0.11 a	0.75 ± 0.48 a	738.6 ± 12.31 a	35.31 ± 17.59 b	24,872 ± 6452 b
QWS	1.05 ± 0.24 a	0.40 ± 0.24 a	564.3 ± 14.85 b	38.50 ± 26.88 b	21,799 ± 9855 b
AC	1.41 ± 0.08 a	1.39 ± 0.18 a	374.0 ± 9.18 c	149.0 ± 23.27 a	55,630 ± 8535 a
AWS	1.38 ± 0.19 a	1.00 ± 0.00 a	350.5 ± 16.03 c	94.26 ± 26.88 ab	32,741 ± 9855 ab
Sig.					
S	*	*	***	**	*
T	NS	NS	***	NS	NS
S × T	NS	NS	***	*	*

## Data Availability

The data that support the findings of this study are available from the corresponding authors, upon reasonable request.
